# Comparison of Road Traffic Injury Characteristics between Local versus Floating Migrant Patients in a Tertiary Hospital between 2007 and 2010

**DOI:** 10.1371/journal.pone.0082640

**Published:** 2014-01-27

**Authors:** Chungui Xu, Yanhua Wang, Na Han, Yuhui Kou, Xiaofeng Yin, Peixun Zhang, Tianbing Wang, Dianying Zhang, Baoguo Jiang

**Affiliations:** Department of Trauma and Orthopaedics, Peking University People's Hospital, Beijing, China; Cardiff University, United Kingdom

## Abstract

**Background:**

The aim of this study is to give a description of the road traffic injuries (RTIs) characteristics of floating migrant population by comparing with those of local residents in a harbor city of China.

**Methods:**

A population-based descriptive study was carried out between 2007 and 2010 with RTI patient records from the Fifth Center Hospital of Tianjin. Inpatient diagnoses of RTI patients were defined using the International Classification of Diseases, Tenth Revision (ICD-10) codes. We analyzed the demographics and general characteristics of RTI patients that were in the hospital during the four years. In order to compare the group differences between local resident patients and floating migrant patients, the distribution of their ages, diagnoses, severity of injuries, duration of inpatient stays, hospitalization cost were analyzed.

**Results:**

People between the ages of 16 and 55 were the most likely to suffer RTIs. The floating migrant patients between the ages of 16 and 45 had a higher incidence of accidents, while local resident patients between 46 and 55 had a higher incidence of accidents. Compared to local resident patients, floating migrant patients were more vulnerable to open injuries and severe traffic injuries. With the severity of injuries ranked from mild to severe, floating migrant patients had lower duration of inpatient stay, but higher hospitalization costs compared to local resident patients.

**Conclusions:**

Floating migrant patients had a different age distribution, severity of injuries, diseases, inpatient duration and hospitalization cost compared with local resident patients. Compared to local resident patients, floating migrants had a higher risk to RTIs and were more vulnerable to severer traffic accidents at lower ages.

## Introduction

Over nearly the last three decades, a new demographic phenomenon in China has attracted increasing attention [1]. The ‘floating population’ (or *liudong renkou* in Chinese) refers to the large and increasing number of migrants without local household registration status (namely *hukou*). According to the report of China National Bureau of Statistics, China had 230 million people in its floating population in 2011, representing 19% of the total population [2]. As a result of the gap between rural and urban incomes, the ‘floating’ migrant population came from the countryside to the cities in pursuit of a better life [3]. This rural-urban migration poses significant challenges, especially for China's welfare system concerning both local and migrant residents. Without local household registration, the floating migrants are not entitled to some of the benefits that local people enjoy. They face daunting problems, particularly with access to healthcare, adequate housing, employment opportunities, pension plans and school enrollment for their children. Recently, the National People's Congress of China has highlighted the plight of the ‘floating population’ and outlined policies for education and basic health services.

Some epidemiological investigation has been done on the health problems of floating population in China, such as some infected diseases [4], [5], unintentional injuries under six years old children [6] and so on. However, the characteristics of road traffic injuries (RTIs) among floating population have never been reported. There may be two reasons. First, such a massive migration of people from undeveloped regions to developed regions was scarcely seen in the other countries of the world except China. Second, most studies about the floating population health were focused on the infected diseases, because the migration of people is closely related to the spread of infected diseases, and the impact of infected diseases is obviously seen. An example is the SARS in 2003 of China [7], [8]. However RTIs could happen on any people independent of the household registration, so research regarding the differences in RTIs, between local resident patients and floating migrant patients, has been scarce. But the floating population of China had a fast increase from 7 million in 1982 to 22 million in 1990 to 79 million in 2000 and 230 million in 2011 [1]. Due to the high mobility characteristics of these people, the fast expansion of floating population could be a factor contributing to the high frequency of road traffic accidents. In China, there were 3.9 million traffic accidents reported in 2010, in which 65 thousand people died and 2.5 million peoples were injured with a direct property loss of around 143 million dollars [9]. A sampling survey of injury deaths in Chinese people also found that, from 1991 to 2005, the proportion of all injury deaths due to traffic accident increased from 15.00% to 33.79% clearly showing a rising trend [10]. So it will make sense to have an investigation on the RTIs characteristics of the floating population that was unclear before.

In this study, we collected the hospital records of RTI patients from the emergency department of the Fifth Center Hospital of Tianjin, and analyzed the RTIs characteristics of floating population in comparison with those of local residents. This hospital is located in the Binhai New District of Tianjin city. It is largest tertiary hospital in this district. More than one-third of the RTI patients in this district were treated in this hospital. Other RTI patients were scattered among the hospitals near the accident spots. As an important harbor in north China, masses of freight were transported from neighboring regions to this district, which lead RTIs here having particular characteristics. This area provides a good sample for research comparing RTIs between local residents and floating population in this district.

## Materials and Methods

### Ethics statement

The study was approved by the Research Ethics Committee at PKUPH and met international biomedical ethics guidelines. Both the Hospital and the Biostatistics Department of Peking University Health Science Center supervised the acquisition of data. The written consent was given by the patients and the next of kin for their information to be stored in the hospital database and used for research.

### Data source

This descriptive study was performed based on the hospital records of RTI patients who suffered from motor vehicle accidents between the beginning of 2007 and the end of 2010. The RTI patients were transferred to the Fifth Center Hospital of Tianjin, by ambulance or by themselves. When patients arrived at the hospital, their personal information and general condition of the road traffic accidents was recorded on an emergency card by the patients themselves or by the accident witnesses. After the initial treatment in the emergency ward, patients were transferred to other departments of this hospital for follow-up treatment. All the emergency patients were treated at best regardless of their household registration. There were no differences for dealing with local resident patients or floating migrant patients. Patient condition was classified as one of the following severity levels according to the examination results: mild, moderate, severe and dead. Traffic accidents sufferers who died before arriving at the hospital were not included, because the persons dead at the scene were not transferred to hospital.

The RTI patients' data were extracted from the medical records database of this hospital. Medical records of all RTI patients were reviewed and diagnosis codes were assigned by trained coders using the International Classification of Diseases 10th Revision (ICD-10). The following database information was provided to the research team: medical record number, date of birth, gender, date of admission, household status, severity of injury, injured parts of the body, admission diagnosis, inpatient diagnoses, ICD-10 diagnoses codes, inpatient duration, hospitalization cost and outcomes at discharge.

### Statistical methods

We used the Excel software to collect the hospital records data and perform the basic statistical analysis. Descriptive statistics included means, median, 25% percentile, 75% percentile, standard deviations, and table and chart design. The parameters observed were age, gender, household status, severity of injury, injured parts of the body, diagnosis, inpatient duration, and hospitalization cost.

## Results

### Demographics and general characteristics

**Figure 1:**
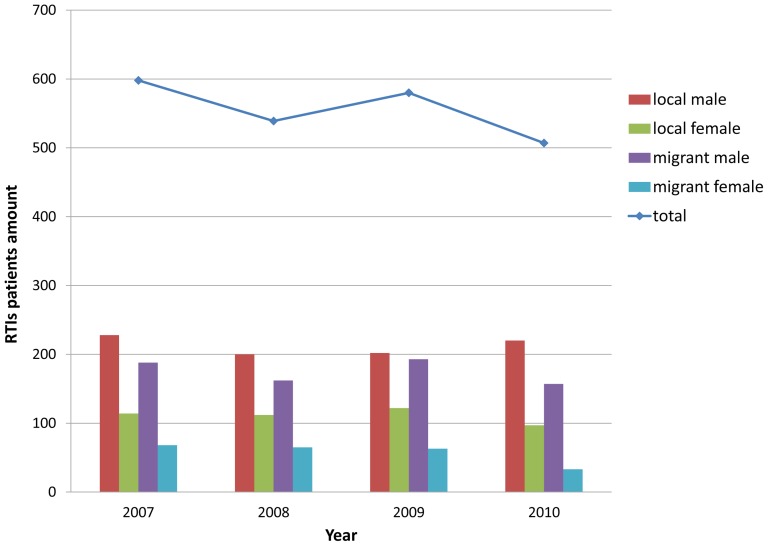
Distribution of male and female RIT patients between 2007 and 2010.

Between 2007 and 2010, there were a total of 2,224 RTI patients transferred to the emergency department of this hospital. The maximum and the minimum number of patients per year were 598 and 507 in 2007 and 2010, respectively. No increasing or decreasing trend in the number of patients per year was found. Patients examined over the four years, included 1,550 male and 674 female patients. The number of male and female patients over the four years is shown in Figure 1. The proportion of female patients of local residents ranged yearly between 30.6% and 37.7%, while that of female patients of floating migrants ranged yearly between 17.4% and 28.6%. There were 1,295 local resident patients and 929 floating migrant patients seen over the four years. The proportion of floating migrant patients was 42.8%, 42.1%, 44.1% and 37.5% for each year of the study. According to the *Tianjin Statistical Yearbooks* [11]–[14], there were 507 thousand, 517 thousand, 529 thousand and 494 thousand people with local household registration in each year from 2007 to 2010. In contrast, the amounts of floating migrants in these four years were 107 thousand, 245 thousand, 272 thousand and 300 thousand people respectively. So the average risks of RTI over the four years for local residents and floating migrants were 0.632 and 1.005 per thousand people.

### Age distribution of RTIs patients

**Figure 2:**
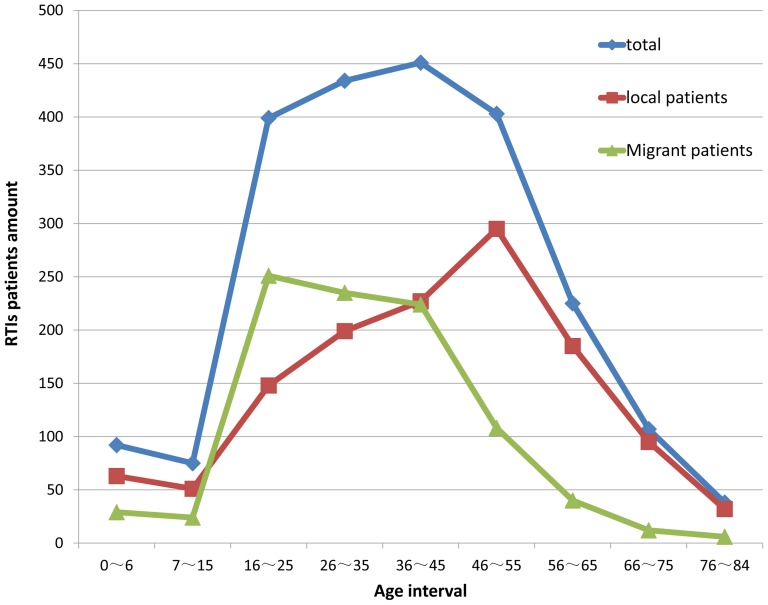
Age distribution of local and floating migrant RTI patients from 2007 to 2010.

The age distribution of local and floating migrant RTI patients over the four years was calculated and is shown in Figure 2. The age range of RTI patients was between 0 and 84 years. Patients under the age of 16 were divided into two groups according to age: preschool age, which was defined as 0 to 6 years old and school age, which was defined as 7 to 15 years old. This is because most children in China go to school at the age of 7 and receive nine years of compulsory education. From 16 to the eldest age, patients were divided into seven groups with each group comprising approximately 10 years. The results showed that, before the age of 16, the preschool age population had a higher distribution of RTIs compared to the school age population. The age distributions of local resident patients and floating migrant patients were also calculated. These distributions were compared and patients were divided into three groups according to age: younger than age 16, age 16 to 45, and age 46 to 84. There were a greater number of local resident patients in the first and the last age groups than floating migrant patients. The number of floating migrant patients was no fewer than that of local resident patients in the 16 to 45 age groups. The peak number of local patients emerged between the ages of 46 and 55, while that of floating migrant patients occurred between the ages of 16 and 45.

### Monthly distribution of RTIs patients

**Figure 3:**
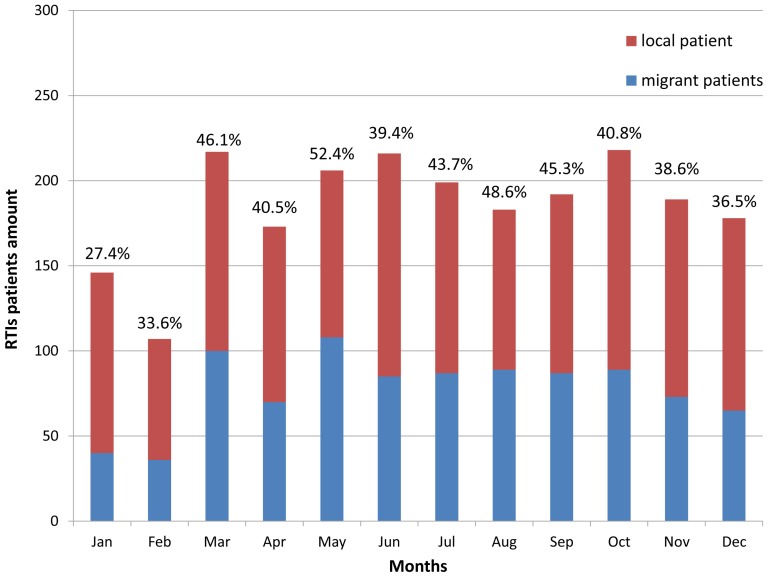
Monthly distribution of RTI patients in the four years from 2007 to 2010 (the data above the columns were proportions of floating migrant population of the 12 months).

Over the 4 years, the number of patients each month was counted and the sum over 12 months was calculated. The results are shown in Figure 3. The 3 months that had the most patients were October (218), March (217) and June (216). In contrast, the 2 months that had the least patients were February (107) and January (146). The proportion of floating migrant patients was also calculated for each month during the 4 years. In January and February, the proportions of floating migrant patients were just 27.4% and 33.6%, which were less than those of other months.

### Distribution of injured regions of the body in traffic accidents

**Figure 4:**
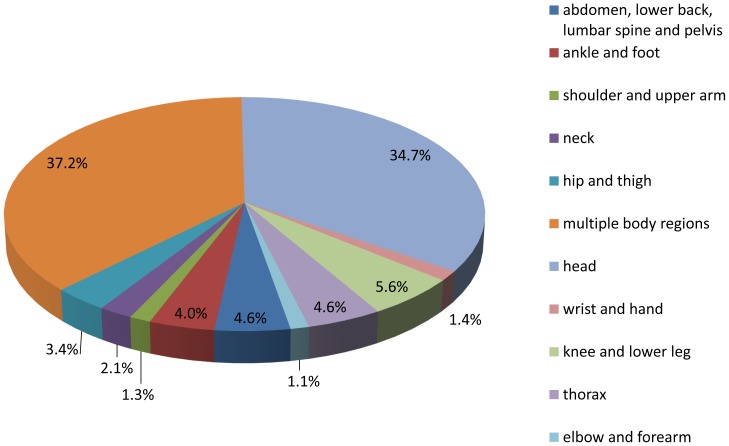
Area of the body injured, as a proportion of total RTIs, from 2007 to 2010.

Information regarding injured regions of the body was registered for all of the patients who were in traffic accidents. Patients were divided into 11 groups according to the injured regions: head, neck, thorax, abdomen, lower back, lumbar spine and pelvis, shoulder and upper arm, elbow and forearm, wrist and hand, hip and thigh, knee and lower leg, ankle and foot, and multiple regions of the body. The proportion of injured regions in RTIs is shown in Figure 4. Most RTI patients had injuries to the head or had injuries to multiple regions of the body. These two groups of patients accounted for 37% and 34.6% of all the patients, respectively. The injury severity and residence of these two groups of patients were analyzed and are shown in Table 1. Except for one patient that died as a result of traumatic rupture of the liver and another patient that died from a sudden cardiac event, patient deaths were caused by head injuries or multiple body region injuries. In the head injury group, the proportions of floating migrant patients classified as mild, moderate and severe were 40.1%, 48.4% and 50% respectively. In the multiple body regions injury group, these proportions were 39.3%, 42.1% and 46.3%, respectively.

**Table 1:** Severity distribution of the head injury group and multiple body regions injury group from 2007 to 2010.

	Head and face injuries				Multiple sites injuries			
severity	mild	moderate	severe	dead	mild	moderate	severe	dead
Local resident patients	251	132	35	13	105	297	65	10
Floating migrant patients	168	124	35	11	68	216	56	6
Proportion of floating migrant patients(%)	40.1	48.4	50.0	45.8	39.3	42.1	46.3	37.5

### Injury characteristics among RTI patients

The hospital diagnoses of all RTI patients were analyzed. In total, 561 types of injuries were diagnosed. Frequently diagnosed injuries were classified as those that occurred no less than 20 times. There were 100 types of injuries that met this criteria. A total of 10 diagnosed injuries involved the head or thorax (e.g. scalp hematoma and rib fracture). These injuries are listed in Table S1 and ranked according to the proportion of floating migrant patients with each injury. The average, median, 25% percentile, 75% percentile and standard deviation of the age of the patients, that had each injury, were also calculated. For some injuries, there were apparent age differences between local and floating migrant patients. For example, the average age of local patients suffered from fracture of pelvis was 47.2±18.1 years old, while that of floating migrant patients was just 33.9±7.6 years old.

Open injuries need more care in the emergency ward because an open wound provides easy access for bacterial infections. The total number of open injuries, among all injuries, was calculated. The result showed that there were 326 local resident patients and 406 floating migrant patients that had open injuries. Table 2 lists the open injuries that occurred in at least 10 cases. The amounts of floating migrant patients were more than those of local resident patients in each of the open injuries except open wound of thigh and elbow, indicating that floating migrant patients were more vulnerable to open injuries.

**Table 2:** Open injuries that occurred at least 10 times from 2007 to 2010.

		Local patients			Migrant patients			
RTIs	T	N	A*	STD*	N	A*	STD*	Prop.
Open wound of ankle	17	4	46.5	27	13	27.6	7.9	0.76
Open wound of auricle Pinna	10	3	33	19.1	7	31.3	12.5	0.70
Open wound of lower leg	45	15	47.5	15.9	30	32.4	10.3	0.67
Open wound of finger(s)	10	4	51.8	19.4	6	29.8	10.7	0.60
Open fracture of tibia	10	4	31.5	20.4	6	32.7	9.4	0.60
Open wound of head	137	57	43.6	14.2	80	33.3	13	0.58
Open fracture of tibial and fibula	36	15	49.7	15.9	21	37.4	12.5	0.58
Open wound of foot	63	27	42.1	22.2	36	28.3	11.8	0.57
Open wound of nose	46	20	31.2	13	26	30	10.9	0.57
Open intracranial injuries	18	8	46.4	19	10	30.1	11.7	0.56
Open wound of ear	39	18	38.3	13.7	21	36.2	10.7	0.54
Open wound of upper arm	13	6	36.8	18.5	7	33.4	16.5	0.54
Open wound of forearm	17	8	40.4	25	9	30.3	6.5	0.53
Open wound of knee	31	15	37.3	11.8	16	31.1	16	0.52
Open wound of lip	106	52	35.7	17	54	31.6	11.7	0.51
Open wound of thigh	21	11	39.6	15.4	10	28.3	4.6	0.48
Open wound of elbow	19	11	39.5	17.1	8	32.5	12.5	0.42

(T = total amount, N = number of local or floating migrant patients, A = average, STD = standard deviation, Prop. = proportion of floating migrant patients with each injury).

* The unit of A and STD is years old.

Fractures that appeared in at least 20 cases were sifted out. These fractures are listed in Table S1 (Lines with yellow background). There were 30 kinds of fractures left after the selection. The ribs and clavicle are the most vulnerable parts of body to fracture and accounted for 11.4% and 5.1% of all traffic injuries, respectively.

### Inpatient duration and hospitalization cost distribution of traffic injury patients

Inpatient duration and hospitalization cost were analyzed for all RTI patients. These patients were grouped according to the severity of injuries and their household status. The average, median, 25% percentile, 75% percentile and standard deviation of inpatient duration and total cost distribution of mild, moderate, severe and deceased groups were calculated and are listed in Table S2. From the mild to severe group, the average, median, 25% percentile, 75% percentile and standard deviation of inpatient duration and total cost increased with the enhancement of severity. In these three groups, the difference in these parameters, between local and floating migrant patients, was more apparent.

## Discussion

Motor vehicle accident is a common cause of road traffic injuries and deaths throughout the world. According to a WHO report, traffic accidents cause 1.27 million deaths and 20 to 50 million injuries around the world annually [15]. In China, with the recent economic boom, vehicle volume and the number of traffic accident fatalities have become the highest in the world. Wang el al. [16]estimated that deaths from vehicle collisions increased 97-fold from 1951 to 1999 in China; and RTIs had become a leading cause of death, accounting for 3.25 percent of all deaths and one third of all injury-related deaths between 2002 and 2006. With such a severe situation in China's road traffic safety, there is an urgent need to study the RTIs characteristics and risk factors that determine traffic violations and traffic accident severity.

A lot of factors contribute to the fast increase of road traffic accidents and injuries in recent China. With the development of the social economy and increased living standard, the ownership of motor vehicles has quickly risen among the middle class population, which has resulted in more road traffic congestion and accidents [17], [18]. Besides, increases in travel between the city and outside is one factor contributing to the high frequency of accidents [19]. In big Chinese cities, such as Beijing and Shanghai, the floating migrants make up a large section of the entire population of the city. Due to the high mobility characteristics of these people, the fast expansion of floating population could be a factor contributing to the high frequency of road traffic accidents. On the other hand, most of the floating migrants were from rural area. They were less educated and involved in low-level physical labor such as freight transportation [1]. A study about the risk factors of traffic accident severity in China showed that driver's gender, education level, household status and occupation were associated with traffic violations and injury severity [20]. So migrant workers exhibited a high risk for severe accidents.

In our study, the RTIs characteristics of floating migrant population were analyzed through comparison with those of local residents. From 2007 to 2010, there were between 500 and 600 RTI patients seen by this hospital annually. Floating migrant patients accounted for 41.6% of RTIs during the four years. This proportion reflected that a great deal of exchange was occurring between this harbor city and outside. The average risks of RTIs over the four years for local residents and floating migrants were different (0.632 versus 1.005 per thousand people). This result indicated that floating population exhibited a higher risk of RTIs compared to local residents. In view of the gender distribution of RTIs patients, the proportion of male patients in these four years ranged between 2/3 and 3/4. Male patients made up the majority of traffic accident sufferers, likely because men are generally the main laborers in society. This phenomenon was more obvious in the floating migrant patients, because female RTIs patients with local household were less than those of floating migrants in each of the four years.

Patients between 16 and 55 years of age constituted the majority of RTI patients likely because people in this age range are the main labor force in society. This was also demonstrated in a recent study examining RTIs in Shanghai [21]. Pre-school children had a higher accident incidence compared to school-children under the age of 16. The probable reason is that compulsory education in China starts in primary school. Before that age, pre-school children are not all taken to the nursery and some children stay at home with parents or grandparents. The age distribution of local and floating migrant patients was also different. Floating migrant patients had a higher accident incidence between ages 16 and 45, while local resident patients had a higher accident incidence between ages 46 and 55. The reason for this difference is likely that the majority of floating migrants in this city are mostly young people who came to the city for business. The detailed age distributions of locals and floating migrant population in this district were not available in any public dataset or yearbook. But according to the report of China National Bureau of Statistics in 2001, among 121 million floating population over the country in 2000, 83% were of working age(from 15 to 64 years). So we believed that the difference spread by age was partly due to different population demographics between local and floating migrant patients. For the local residents, increased age was related to the likelihood of injury from a traffic accident [22].

March, June and October were found to be the months with the most RTI patients with 217, 216 and 218 patients for each month, respectively. February and January were the months that had fewer RTI patients with 107 and 146 patients, respectively. The proportions of floating migrant patients were just 27.4% and 33.6% in these two months, which were less than those of other months too. This is because January and February are usually the months in which the lunar calendar Spring Festival of China occurs. Most floating migrants go back home, thus there are fewer RTIs during these two months. This monthly distribution result is also in accordance with that found in Beijing from 2004 to 2008 [23], which is near Tianjin.

In a traffic accident, any part of body may be injured. The data in this paper showed that most patients had head or multiple body region injuries. These two groups accounted for 34.6% and 37% of all patients. The numbers of local resident patients and floating migrant patients in these two groups were counted and classified according to the severity of injuries: mild, moderate, severe and deceased. The proportion of floating migrant patients increased as the severity of injury increased from mild to severe. We supposed that this was due to the fact that floating migrant patients likely traverse, not only the city district, but also between the city and outside. On the highways, the speed limit is over that of city roads and there is no traffic regulation. Accidents that happen on the highway usually cause severe injuries or death [24]. Meanwhile, many floating migrants have lower level of education. They lack the knowledge and consciousness of safe-driving. This is also a risk factor for traffic violation and accident severity. Therefore, floating migrants are more vulnerable to severe RTIs compared with local residents.

Statistical analysis of RTIs means a lot to emergency clinics. Injuries that had occurred at least 20 times were reviewed. For each of these injuries, we calculated the floating migrant patient proportion with each injury, and the mean, median, 25% percentile and 75% percentile ages in both the local resident patient and floating migrant patient groups. The results showed that the top injuries with higher proportion of floating migrant patients were mostly associated with high energy accidents, for example traumatic spleen rupture, fracture of acetabulum, contusion of kidney or fracture of the shaft of the femur. In contrast, the injuries with smaller proportions were generally upper extremity or neck injuries, or injuries or diseases associated with aging, for example fracture of humerus, superficial injury of neck, hypertension and type 2 diabetes. Among these injuries, the open injuries, except open wound of thigh and open wound of elbow, had the proportions more than 0.5, which indicated that floating migrant patients were more vulnerable to open injuries. For fractures, those with larger proportions were usually associated with high-energy crashes. However, fractures with smaller proportions were usually associated with low-energy crashes or with aging, such as fracture of lumbar vertebra and intertrochanteric fracture of the femur. To sum up, statistical analysis results of RTIs supports that local resident patients are usually associated with low-energy accidents while floating migrant patients are usually associated with high-energy accidents, and that ages have an impact on the RTI distribution among local resident patients.

Due to the reasons mentioned above, inpatient duration and hospitalization costs were different for local versus floating migrant patients. Hospitalization costs for floating migrant patients increased with the severity of injury (mild to severe) from below to a great deal above that of local residents. For the inpatient duration, there was an increase in both the local residents and floating migrants with injury, but that it increased more slowly among floating migrants.

## Conclusion

In the current paper the hospital records of RTI patients seen in a tertiary hospital in a harbor city of China between the year of 2007 and 2010, were analyzed. The results showed that floating migrant patients had different characteristics than local resident patients in terms of age, the type and severity of injuries, inpatient duration, hospitalization cost. We attribute this to the fact that most floating migrants are young businessmen from out of the city and they spend more time on the road, especially on the highway. In contrast, the population of local residents consists of different ages and elder people who are more vulnerable to injuries resulting from traffic accidents compared to younger people. In light of this study, further research should be done and more consideration given to the health of floating migrants.

## Supporting Information

Table S1 **Age distribution of diagnosed injuries that occurred at least 20 times among all patients.** (T = total number of patients in each disease, N = the number of local or migrant patients in each disease, A = average, M = median, Q = 25% percentile, 3Q = 75% percentile, S = standard deviation) * The unit of A, M, Q, 3Q and S is years old.
(DOCX)

Table S2 **Inpatient duration and total cost distribution for mild, moderate, severe injury and deceased groups for both local and floating migrant patients.** * The unit of duration is day. ** The unit of expense is USD.
(DOCX)
