# Supplementary material for: Comparison of Road Traffic Injury Characteristics between Local versus Floating Migrant Patients in a Tertiary Hospital between 2007 and 2010
Source: PLoS One. 2014 Jan 27;9(1):e82640. doi: 10.1371/journal.pone.0082640 (PMC3903469; doi:10.1371/journal.pone.0082640)
Supplement: Table S1 — Age distribution of diagnosed injuries that occurred at least 20 times among all patients. (T = total number of patients in each disease, N = the number of local or migrant patients in each disease, A = average, M = median, Q = 25% percentile, 3Q = 75% percentile, S = standard deviation) * The unit of A, M, Q, 3Q and S is years old. (DOCX) [file pone.0082640.s001.docx]

Table S1. Age distribution of diagnosed injuries that occurred at least 20 times among all patients.

|  |  | **Local resident patients** | | | | | | **Floating migrant patients** | | | | | | **Migrant patients proportion(%)** |
| --- | --- | --- | --- | --- | --- | --- | --- | --- | --- | --- | --- | --- | --- | --- |
| **Name of disease** | **T** | **N** | **A*** | **M*** | **Q*** | **3Q*** | **SD*** | **N** | **A*** | **M*** | **Q*** | **3Q*** | **SD*** |  |
| *Multiple superficial injuries of head* | 22 | 5 | 28.2 | 28 | 22.3 | 35.0 | 6.8 | 17 | 32.9 | 33 | 22.8 | 38.3 | 12.3 | 77.3 |
| *Open wound of lower leg* | 45 | 15 | 47.5 | 46 | 40.3 | 57.8 | 15.9 | 30 | 32.4 | 31.5 | 23.0 | 40.0 | 10.3 | 66.7 |
| *Rupture of spleen* | 33 | 11 | 38.9 | 40 | 28.3 | 51.0 | 17.9 | 22 | 29.9 | 26.5 | 20.0 | 36.0 | 11.6 | 66.7 |
| *Fracture of acetabulum* | 24 | 8 | 38.4 | 38 | 29.0 | 47.0 | 9.3 | 16 | 43.0 | 41 | 35.0 | 51.5 | 13.1 | 66.7 |
| *Contusion of kidney* | 29 | 10 | 35.6 | 34.5 | 24.0 | 51.0 | 15.3 | 19 | 30.1 | 26 | 20.8 | 42.3 | 15.4 | 65.5 |
| *Fracture of shaft of femur* | 48 | 17 | 32.9 | 31 | 18.3 | 44.5 | 20.3 | 31 | 30.4 | 26 | 21.8 | 38.0 | 11.2 | 64.6 |
| *Open wound of eyelid* | 100 | 38 | 41.8 | 41 | 27.0 | 53.0 | 15.8 | 62 | 33.4 | 32 | 24.0 | 41.0 | 11.5 | 62.0 |
| *Fracture of skull* | 73 | 30 | 43.5 | 47 | 34.0 | 56.0 | 17.6 | 43 | 34.7 | 35 | 24.0 | 44.0 | 14.4 | 58.9 |
| *Open wound of head* | 137 | 57 | 43.6 | 44 | 33.8 | 52.0 | 14.2 | 80 | 33.3 | 32 | 23.0 | 41.5 | 13.0 | 58.4 |
| *Open fracture of both tibia and fibula* | 36 | 15 | 49.7 | 52 | 40.3 | 60.0 | 15.9 | 21 | 37.4 | 35 | 28.0 | 44.0 | 12.5 | 58.3 |
| *Open wound of foot* | 63 | 27 | 42.1 | 46 | 26.8 | 58.8 | 22.2 | 36 | 28.3 | 28 | 21.5 | 36.5 | 11.8 | 57.1 |
| *Open wound of nose* | 46 | 20 | 31.2 | 33 | 22.0 | 39.0 | 13.0 | 26 | 30.0 | 30 | 23.0 | 34.0 | 10.9 | 56.5 |
| *Fracture of nasal bones* | 39 | 17 | 42.9 | 43 | 30.8 | 53.5 | 15.9 | 22 | 28.5 | 24 | 19.0 | 39.0 | 15.1 | 56.4 |
| *Open wound of scalp* | 323 | 148 | 41.5 | 41.5 | 31.0 | 53.0 | 15.6 | 175 | 34.6 | 34 | 24.0 | 44.0 | 14.0 | 54.2 |
| *Fracture of temporal bone* | 20 | 9 | 41.4 | 45 | 35.5 | 52.8 | 17.1 | 11 | 30.5 | 30 | 25.0 | 33.5 | 8.7 | 55.0 |
| *Fracture of cervical* | 31 | 14 | 39.6 | 36 | 30.0 | 44.0 | 14.7 | 17 | 38.9 | 41 | 27.8 | 45.3 | 13.0 | 54.8 |
| *Multiple injuries of head* | 22 | 10 | 33.7 | 32.5 | 26.0 | 42.0 | 15.8 | 12 | 29.6 | 25.5 | 22.0 | 37.5 | 13.0 | 54.5 |
| *Fracture of base of skull* | 91 | 42 | 43.3 | 44 | 37.0 | 53.0 | 15.7 | 49 | 32.1 | 30 | 23.0 | 40.3 | 13.0 | 53.8 |
| *Open wound of ear* | 39 | 18 | 38.3 | 39.5 | 25.0 | 50.0 | 13.7 | 21 | 36.2 | 33 | 29.3 | 41.3 | 10.7 | 53.8 |
| *Traumatic extradural hemorrhage* | 69 | 32 | 39.8 | 42.5 | 28.5 | 53.0 | 16.6 | 37 | 34.3 | 32 | 25.8 | 41.5 | 10.0 | 53.6 |
| *Contusion of abdomen* | 136 | 64 | 39.4 | 39.5 | 28.0 | 51.0 | 17.6 | 72 | 31.4 | 30 | 23.5 | 41.0 | 13.6 | 52.9 |
| *Open wound of face* | 199 | 94 | 36.8 | 35.5 | 26.0 | 46.0 | 15.3 | 105 | 32.8 | 32 | 22.8 | 41.0 | 14.0 | 52.8 |
| *Contusion of lumbar spine and pelvis* | 56 | 27 | 46.4 | 48 | 37.3 | 58.8 | 17.1 | 29 | 34.6 | 33 | 23.8 | 40.3 | 14.4 | 51.8 |
| *Fracture of thoracic vertebra* | 31 | 15 | 49.0 | 51 | 42.3 | 57.0 | 12.1 | 16 | 34.6 | 32.5 | 21.5 | 48.0 | 14.5 | 51.6 |
| *Open wound of knee* | 31 | 15 | 37.3 | 39 | 31.3 | 44.8 | 11.8 | 16 | 31.1 | 25.5 | 22.5 | 39.0 | 16.0 | 51.6 |
| *Open wound of lip* | 106 | 52 | 35.7 | 34.5 | 25.0 | 47.0 | 17.0 | 54 | 31.6 | 30 | 22.0 | 38.0 | 11.7 | 50.9 |
| *Open wound of hand* | 26 | 13 | 47.6 | 42 | 33.8 | 73.8 | 22.4 | 13 | 36.2 | 35 | 28.5 | 43.3 | 9.1 | 50.0 |
| *Fracture of pubis* | 26 | 13 | 43.8 | 48 | 32.8 | 56.3 | 15.3 | 13 | 29.8 | 30 | 20.3 | 36.5 | 15.1 | 50.0 |
| *Sprain involving bitial collateral*  *Ligament of knee* | 24 | 12 | 45.5 | 50 | 32.5 | 54.5 | 17.3 | 12 | 32.4 | 29 | 26.0 | 38.0 | 8.4 | 50.0 |
| *Traumatic subarachnoid hemorrhage* | 223 | 112 | 46.2 | 48 | 35.5 | 57.0 | 16.7 | 111 | 35.1 | 35 | 23.3 | 43.8 | 13.8 | 49.8 |
| *Fracture of lateral malleolus* | 31 | 16 | 53.1 | 56.5 | 47.0 | 61.0 | 11.8 | 15 | 34.7 | 35 | 22.5 | 45.5 | 16.6 | 48.4 |
| *Diffuse brain injury* | 185 | 96 | 48.6 | 50 | 36.0 | 60.0 | 16.4 | 89 | 36.1 | 37 | 23.0 | 44.3 | 14.4 | 48.1 |
| *Tear of meniscus, current* | 21 | 11 | 42.3 | 48 | 26.5 | 54.0 | 15.1 | 10 | 35.6 | 34 | 28.0 | 44.0 | 8.3 | 47.6 |
| *Open wound of thigh* | 21 | 11 | 39.6 | 46 | 29.5 | 52.5 | 15.4 | 10 | 28.3 | 28 | 25.0 | 31.0 | 4.6 | 47.6 |
| *Multiple superficial injuries of forearm* | 36 | 19 | 43.7 | 41 | 29.3 | 55.3 | 14.8 | 17 | 24.4 | 26 | 18.8 | 30.8 | 10.9 | 47.2 |
| *Multiple superficial injuries of lower leg* | 34 | 18 | 41.1 | 38 | 27.0 | 52.0 | 15.2 | 16 | 29.5 | 30.5 | 20.5 | 37.5 | 11.7 | 47.1 |
| *Fracture of pelvis* | 49 | 26 | 47.2 | 49 | 31.0 | 58.0 | 18.1 | 23 | 33.9 | 35 | 27.0 | 40.8 | 7.6 | 46.9 |
| *Contusion of ankle* | 47 | 25 | 36.9 | 38 | 23.0 | 48.3 | 17.8 | 22 | 33.5 | 35 | 23.0 | 42.0 | 12.2 | 46.8 |
| *Fracture of both tibia and fibula* | 58 | 31 | 40.6 | 47 | 34.0 | 55.5 | 19.5 | 27 | 31.9 | 33 | 25.3 | 38.8 | 10.1 | 46.6 |
| *Fracture of superior maxilla* | 41 | 22 | 41.9 | 42.5 | 29.0 | 53.0 | 16.6 | 19 | 38.0 | 40 | 29.0 | 48.3 | 12.5 | 46.3 |
| *Superficial injury of ankle and foot, part*  *Unspecified* | 40 | 22 | 38.5 | 46.5 | 13.0 | 56.0 | 22.4 | 18 | 26.1 | 25.5 | 10.0 | 38.0 | 17.9 | 45.0 |
| *Fracture of zygomatic arch* | 29 | 16 | 48.7 | 52 | 41.5 | 56.5 | 14.1 | 13 | 34.9 | 37 | 25.5 | 42.8 | 11.4 | 44.8 |
| *Traumatic subdural hemorrhage* | 112 | 62 | 51.5 | 53 | 39.0 | 65.0 | 17.0 | 50 | 35.7 | 36 | 24.0 | 43.0 | 13.7 | 44.6 |
| *Contusion of lung* | 182 | 101 | 49.4 | 50 | 42.8 | 58.3 | 13.2 | 81 | 36.8 | 36 | 25.0 | 44.5 | 13.6 | 44.5 |
| *Contusion of nose* | 52 | 29 | 39.4 | 37 | 23.0 | 56.5 | 22.1 | 23 | 29.0 | 24 | 19.3 | 40.8 | 13.7 | 44.2 |
| *Fracture of scapula* | 34 | 19 | 49.2 | 53 | 35.5 | 58.5 | 14.5 | 15 | 41.8 | 43 | 31.3 | 52.3 | 15.9 | 44.1 |
| *Dislocation of tooth (traumatic)* | 66 | 37 | 43.8 | 41 | 30.5 | 57.0 | 18.1 | 29 | 28.5 | 26 | 19.0 | 37.0 | 12.5 | 43.9 |
| *Superficial injury of scalp* | 112 | 63 | 42.5 | 42 | 25.3 | 56.0 | 19.2 | 49 | 34.0 | 34 | 23.8 | 42.3 | 16.1 | 43.8 |
| *Intracranial pneumatocele* | 23 | 13 | 43.1 | 42 | 33.5 | 52.3 | 16.0 | 10 | 32.2 | 31 | 25.0 | 39.0 | 12.2 | 43.5 |
| *Superficial injuries involving multiple*  *regions of lower limb(s)* | 30 | 17 | 41.9 | 51 | 28.3 | 55.0 | 18.4 | 13 | 31.6 | 30 | 24.8 | 38.5 | 11.9 | 43.3 |
| *Traumatic hemothorax* | 37 | 21 | 49.8 | 51 | 39.5 | 59.5 | 13.4 | 16 | 40.3 | 40.5 | 33.5 | 45.0 | 9.8 | 43.2 |
| *Abrasion of hand* | 86 | 49 | 40.3 | 44 | 20.8 | 56.0 | 19.8 | 37 | 31.2 | 26 | 21.0 | 38.3 | 14.6 | 43.0 |
| *Traumatic intracranial injury* | 104 | 60 | 46.4 | 46.5 | 33.5 | 58.5 | 15.6 | 44 | 38.4 | 38 | 29.0 | 46.0 | 13.4 | 42.3 |
| *Traumatic hemopneumothorax* | 38 | 22 | 48.3 | 48 | 33.0 | 62.0 | 17.4 | 16 | 41.9 | 43.5 | 29.5 | 54.0 | 14.8 | 42.1 |
| *Fracture of orbit* | 50 | 29 | 43.4 | 43 | 29.5 | 54.3 | 15.9 | 21 | 37.3 | 39 | 30.8 | 46.5 | 12.4 | 42.0 |
| *Traumatic pneumothorax* | 24 | 14 | 39.4 | 45.5 | 25.0 | 54.0 | 16.6 | 10 | 30.2 | 31 | 20.0 | 40.0 | 9.2 | 41.7 |
| *Contusion of scalp* | 84 | 49 | 40.0 | 39 | 28.5 | 54.5 | 19.6 | 35 | 29.9 | 30 | 22.0 | 34.8 | 15.8 | 41.7 |
| *Fracture of tibia* | 77 | 45 | 42.7 | 47 | 27.5 | 56.0 | 18.6 | 32 | 35.7 | 35 | 26.0 | 46.5 | 13.7 | 41.6 |
| *Contusion of elbow* | 123 | 72 | 42.7 | 45.5 | 26.0 | 56.5 | 18.3 | 51 | 33.7 | 34 | 24.3 | 42.0 | 14.0 | 41.5 |
| *Contusion of eye* | 81 | 48 | 45.0 | 44.5 | 34.5 | 53.5 | 14.6 | 33 | 36.8 | 37 | 24.0 | 45.0 | 14.7 | 40.7 |
| *Abrasion of knee* | 107 | 64 | 38.3 | 37 | 24.0 | 54.5 | 18.2 | 43 | 28.3 | 29 | 22.0 | 36.5 | 12.2 | 40.2 |
| *Fracture of clavicle* | 113 | 68 | 41.6 | 45.5 | 27.5 | 53.0 | 17.1 | 45 | 34.8 | 35 | 26.5 | 42.0 | 12.2 | 39.8 |
| *Fracture of fibula* | 68 | 41 | 51.3 | 54 | 40.8 | 62.0 | 17.3 | 27 | 35.8 | 36 | 22.0 | 44.3 | 16.1 | 39.7 |
| *Fracture of radius* | 33 | 20 | 45.9 | 49 | 36.0 | 59.5 | 19.3 | 13 | 37.8 | 34 | 31.5 | 44.5 | 11.0 | 39.4 |
| *Contusion of lower leg* | 66 | 40 | 46.8 | 49 | 37.5 | 57.0 | 16.4 | 26 | 29.4 | 26 | 18.0 | 41.0 | 18.0 | 39.4 |
| *Fracture of rib* | 254 | 154 | 48.2 | 49 | 39.0 | 58.0 | 13.6 | 100 | 41.3 | 41 | 33.5 | 51.5 | 13.4 | 39.4 |
| *Unspecified injury of lower back* | 87 | 53 | 48.0 | 49 | 35.8 | 63.0 | 18.3 | 34 | 35.0 | 35 | 25.0 | 44.0 | 13.8 | 39.1 |
| *Hydrothorax* | 82 | 50 | 47.1 | 50 | 36.0 | 56.0 | 14.1 | 32 | 40.2 | 39 | 26.5 | 52.5 | 16.7 | 39.0 |
| *Fracture of patella* | 26 | 16 | 37.7 | 40.5 | 30.0 | 48.0 | 12.1 | 10 | 34.1 | 33.5 | 27.0 | 43.0 | 10.8 | 38.5 |
| *Rupture of cruciate ligament* | 21 | 13 | 39.8 | 43 | 28.8 | 50.0 | 11.8 | 8 | 31.5 | 27.5 | 27.0 | 34.5 | 7.6 | 38.1 |
| *Abrasion of face, NOS* | 161 | 100 | 35.5 | 36 | 22.0 | 48.5 | 18.1 | 61 | 30.3 | 32 | 20.0 | 39.0 | 13.7 | 37.9 |
| *Contusion of face, NOS* | 134 | 84 | 40.7 | 41.5 | 25.0 | 54.0 | 18.7 | 50 | 30.0 | 31 | 22.0 | 38.0 | 12.3 | 37.3 |
| *Contusion of chest wall* | 366 | 230 | 44.6 | 45 | 34.0 | 56.0 | 15.1 | 136 | 36.6 | 35.5 | 24.0 | 45.0 | 13.9 | 37.2 |
| *Fracture of lumbar vertebra* | 84 | 53 | 50.3 | 54 | 40.0 | 61.0 | 16.4 | 31 | 39.6 | 40 | 29.0 | 47.5 | 15.1 | 36.9 |
| *Superficial injury of lip and oral cavity* | 33 | 21 | 28.9 | 25 | 15.5 | 50.0 | 20.6 | 12 | 32.6 | 33 | 25.5 | 40.0 | 9.3 | 36.4 |
| *Fracture of medial malleolus* | 36 | 23 | 46.2 | 51 | 33.5 | 55.0 | 15.3 | 13 | 30.5 | 34 | 21.5 | 37.8 | 11.0 | 36.1 |
| *Conjunctival hemorrhage* | 25 | 16 | 40.0 | 40 | 24.0 | 54.0 | 19.2 | 9 | 33.1 | 34 | 23.8 | 40.5 | 11.3 | 36.0 |
| *Fracture of other toe* | 31 | 20 | 44.8 | 51.5 | 31.0 | 58.0 | 20.0 | 11 | 29.4 | 28 | 21.3 | 35.0 | 10.1 | 35.5 |
| *Contusion of foot* | 51 | 33 | 36.1 | 34 | 20.0 | 52.5 | 22.3 | 18 | 26.6 | 28.5 | 21.0 | 34.0 | 9.4 | 35.3 |
| *Hematoma of scalp* | 520 | 339 | 42.0 | 44 | 26.0 | 55.8 | 18.6 | 181 | 34.1 | 32 | 22.8 | 45.0 | 14.7 | 34.8 |
| *Superficial injuries of head, part*  *Unspecified* | 409 | 268 | 40.8 | 41.5 | 25.0 | 55.0 | 19.8 | 141 | 31.9 | 32 | 22.0 | 39.3 | 13.4 | 34.5 |
| *Contusion of thigh* | 38 | 25 | 36.6 | 32 | 22.0 | 51.8 | 21.7 | 13 | 38.7 | 34 | 22.0 | 59.0 | 20.8 | 34.2 |
| *Contusion of shoulder* | 122 | 82 | 41.7 | 42 | 26.0 | 56.0 | 18.2 | 40 | 33.8 | 32.5 | 23.0 | 41.0 | 13.5 | 32.8 |
| *Dislocation of acromioclavicular joint* | 25 | 17 | 45.3 | 50 | 33.5 | 60.0 | 20.4 | 8 | 39.6 | 40.5 | 29.0 | 50.0 | 11.7 | 32.0 |
| *Fracture of metatarsal bone* | 55 | 38 | 47.1 | 49.5 | 34.0 | 59.0 | 19.4 | 17 | 32.8 | 31 | 20.0 | 45.0 | 18.2 | 30.9 |
| *Contusion of back* | 43 | 30 | 39.5 | 46 | 20.0 | 57.0 | 21.6 | 13 | 31.7 | 32 | 21.8 | 48.3 | 17.5 | 30.2 |
| *Fracture of transtrochanteric section*  *of femur* | 20 | 14 | 63.0 | 63 | 57.0 | 71.0 | 10.1 | 6 | 42.0 | 40.5 | 39.0 | 44.0 | 6.1 | 30.0 |
| *Contusion of eyelid* | 97 | 68 | 49.0 | 50.5 | 35.5 | 60.0 | 17.7 | 29 | 30.9 | 33 | 22.0 | 37.3 | 9.9 | 29.9 |
| *Superficial injury of nose* | 27 | 19 | 41.7 | 39 | 26.8 | 58.8 | 20.2 | 8 | 38.8 | 39.5 | 31.5 | 44.5 | 10.1 | 29.6 |
| *Contusion of knee* | 153 | 108 | 43.0 | 43 | 28.5 | 55.0 | 19.1 | 45 | 31.1 | 27 | 21.0 | 38.3 | 12.9 | 29.4 |
| *Contusion of hand* | 59 | 42 | 45.4 | 48 | 31.0 | 59.0 | 17.7 | 17 | 36.7 | 38 | 30.3 | 46.0 | 15.2 | 28.8 |
| *Fracture of ulna* | 21 | 15 | 50.9 | 55 | 42.8 | 65.8 | 23.0 | 6 | 34.7 | 39 | 23.0 | 41.0 | 10.6 | 28.6 |
| *Contusion of hip* | 110 | 81 | 43.4 | 47 | 29.0 | 56.5 | 18.3 | 29 | 32.1 | 29 | 24.0 | 37.8 | 12.8 | 26.4 |
| *Hypertension* | 23 | 17 | 57.1 | 60 | 47.0 | 68.3 | 13.7 | 6 | 48.8 | 48 | 42.0 | 53.0 | 11.1 | 26.1 |
| *Superficial injuries involving multiple*  *regions of upper limb(s) with lower limb(s)* | 28 | 21 | 33.8 | 33 | 17.8 | 50.5 | 19.5 | 7 | 43.0 | 42 | 34.3 | 48.3 | 17.7 | 25.0 |
| *Fracture of humerus* | 31 | 24 | 42.7 | 49 | 24.5 | 57.0 | 22.0 | 7 | 33.1 | 29 | 26.5 | 36.5 | 9.0 | 22.6 |
| *Multifacial superficial injuries of neck* | 52 | 41 | 42.1 | 39 | 31.8 | 50.3 | 13.6 | 11 | 34.0 | 30 | 22.5 | 40.8 | 16.7 | 21.2 |
| *Superficial injury of neck, part unspecified* | 89 | 70 | 41.6 | 42 | 30.0 | 51.0 | 15.7 | 19 | 34.9 | 32 | 23.3 | 38.5 | 14.1 | 21.3 |
| *Injury of nerve root of cervical spine* | 25 | 22 | 44.7 | 43.5 | 34.0 | 54.0 | 13.4 | 3 | 32.3 | 27 | 25.5 | 40.5 | 11.0 | 12.0 |
| *Type 2 diabetes mellitus* | 26 | 24 | 55.9 | 55 | 47.0 | 62.0 | 12.0 | 2 | 54.0 | 54 | 44.0 | 64.0 | 14.1 | 7.7 |

(T=total number of patients in each disease, N=the number of local or migrant patients in each disease, A=average, M=median, Q=25% percentile, 3Q=75% percentile, S=standard deviation)

* The unit of A, M, Q, 3Q and S is years old
