# Supplementary material for: Comparison of Road Traffic Injury Characteristics between Local versus Floating Migrant Patients in a Tertiary Hospital between 2007 and 2010
Source: PLoS One. 2014 Jan 27;9(1):e82640. doi: 10.1371/journal.pone.0082640 (PMC3903469; doi:10.1371/journal.pone.0082640)
Supplement: Table S2 — Inpatient duration and total cost distribution for mild, moderate, severe injury and deceased groups for both local and floating migrant patients. * The unit of duration is day. ** The unit of expense is USD. (DOCX) [file pone.0082640.s002.docx]

Table S2. Inpatient duration and total cost distribution for mild, moderate, severe injury and deceased groups for both local and floating migrant patients

| Severity | Residence |  | Mean | Median | 25% percentile | 75% percentile | Standard deviation |
| --- | --- | --- | --- | --- | --- | --- | --- |
| Mild | Local | Duration* | 9.13 | 7 | 4 | 11 | 9.6 |
|  |  | Expense** | 844.26 | 484.72 | 235.09 | 1028.65 | 1403.35 |
|  | Migrant | Duration* | 9.31 | 7 | 4 | 12 | 8.86 |
|  |  | Expense** | 784.97 | 541.87 | 265.74 | 1003.74 | 955.19 |
| Moderate | Local | Duration* | 19.19 | 14 | 8 | 24 | 18.76 |
|  |  | Expense** | 2101.01 | 1047.44 | 455.11 | 2607.20 | 2717.93 |
|  | Migrant | Duration* | 16.77 | 12 | 7 | 20 | 17.99 |
|  |  | Expense** | 2166.28 | 1115.17 | 514.08 | 2731.79 | 3121.35 |
| Severe | Local | Duration* | 39.6 | 29 | 9 | 55 | 41.24 |
|  |  | Expense** | 5670.27 | 2217.05 | 925.75 | 7508.37 | 7409.60 |
|  | Migrant | Duration* | 31.66 | 23.5 | 12 | 43 | 27.74 |
|  |  | Expense** | 6728.20 | 4139.38 | 1544.32 | 9465.84 | 7037.66 |
| Deceased | Local | Duration* | 10.38 | 4 | 1 | 6 | 32.82 |
|  |  | Expense** | 3823.20 | 1954.46 | 707.32 | 4029.51 | 5793.80 |
|  | Migrant | Duration* | 5.78 | 2 | 1 | 4 | 13.89 |
|  |  | Expense** | 2074.46 | 934.81 | 577.25 | 1880.06 | 3925.95 |

* The unit of duration is day

** The unit of expense is USD
